# Advances in reproductive biotechnologies

**DOI:** 10.14202/vetworld.2016.388-395

**Published:** 2016-04-18

**Authors:** K. K. Choudhary, K. M. Kavya, A. Jerome, R. K. Sharma

**Affiliations:** 1 ICAR-National Dairy Research Institute, Karnal - 132 001, Haryana, India; 2 ICAR-Indian Veterinary Research Institute, Bareilly - 243 122, Uttar Pradesh, India; 3 ICAR-Central Institute for Research on Buffaloes, Hisar - 125 001, Haryana, India

**Keywords:** assisted reproductive technologies, biotechnology, livestock, reproduction

## Abstract

In recent times, reproductive biotechnologies have emerged and started to replace the conventional techniques. It is noteworthy that for sustained livestock productivity, it is imperative to start using these techniques for facing the increasing challenges for productivity, reproduction and health with impending environment conditions. These recent biotechniques, both in male and female, have revolutionized and opened avenues for studying and manipulating the reproductive process both *in vitro* and *in vivo* in various livestock species for improving tis efficiency. This review attempts to highlight pros and cons, on the recent developments in reproductive biotechnologies, both in male and female in livestock species.

## Introduction

Efficient reproductive performance and monitoring are imperative for sustainability in any livestock production system, especially for milk, meat, draft, and replacement animals. In recent times, there has been increasing challenges for increasing productivity and disease with altering climate. These targets, thought to some extent, can be achieved by conventional reproduction techniques. Advent and use of modern reproductive technologies have opened many avenues to study, treat and manipulate the reproductive phenomenon both *in vitro* and *in vivo* to improve reproductive performance in various domestic species of livestock. The various developments in the field of reproductive biotechnologies are discussed below.

## Semen Sexing

This technology is used for producing offspring of the desired sex, either male or female. Selecting the sex of the progeny using sex-sorted sperm has been an advantage for animal breeders [1,2]. This technique works on the principle of flow cytometric separation of fluorescent-labeled X-chromosome bearing spermatozoa from the sperms carrying fluorescent-labeled Y-chromosome. At present, this technology is capable of analyzing over 100,000 events (sperms) per second and can sort 70,000 events (sperms) per second. By this way it is capable of sorting 15 million spermatozoa per hour into X- and Y-bearing sperms [3] and accuracy of predicting the sex of calves is between 85% and 95% accurate [4]. This technique has been used in various domestic species including buffaloes [5-8]. Although the number of sorted sperm tends to be low, acceptable pregnancy rates have been obtained by *in vivo* by deep intrauterine insemination [9,10]. In addition, semen sexing can be used for enhancing progeny testing program, increase breeding male production, reduce the incidence of sex-linked diseases, besides conservation of superior and rare animals. One of the main limitations of this technique is the low number of sexed sperm produced per unit of time, and sexed sperm display a variety of damages, *viz*., destabilization of sperm membrane and capacitation-like changes thereby reducing lifespan of sorted spermatozoa in the female genital tract [11,12]. However, new generation flow cytometer with high sorting rates have opened avenues for increasing sorted sperm output with minimal or no damage to sperm.

## Sperm Encapsulation

This involves encapsulation of sperm for longer preservation of sperm *in vivo*. This technology was designed to extend the life of spermatozoa at body temperature, and to allow progressive release of viable spermatozoa over several days in various domestic species including human. Bovine sperm microencapsulation is done using capsules of components, *viz*., calcium alginate, cellulose sulfate-poly-diallyl-dimethyl-ammonium chloride (CS-pDADMAC), poly-l-lysine, polyvinylamine and protamine sulfate membrane using standard encapsulation procedure. Usually, the size of the sperm capsule varies between 0.75 and 1.5 mm with sperm concentrations of 45-180 million per ml. Studies revealed that bovine spermatozoa showed high motility rates within CS-pDADMAC based capsules as compared to other polymers. For sperm encapsulation extenders, *viz*., CUE, CAPROGEN and egg yolk-citrate-glycerol have been used without any drastic effects on sperm. Thus, encapsulation helps not only in sustained release of sperm as well as prevents cryocapacitation and also reported to have increased conception rate [13]. Although this technique has been developed in cattle and swine, still it needs more sophisticated instrument for encapsulation and standardization, to be used under field conditions in other livestock species.

## Sperm Transcriptomics

Transcriptomics involves the study of mRNA at various developmental stages including spermatogenesis. Sperm deliver paternal genes to the oocyte and also carry remnant messenger RNA arising out of spermatogenesis. These transcripts are associated with different cellular and biological processes. Profiling of these transcripts using microarrays or by next generation sequencing technologies has proved highly effective tool for studying sperm mRNA expression profile analysis and polymorphism in related genes. Studies have shown that there is a difference of expression profile transcripts in high-fertility bulls with higher concentrations of transcripts for membrane and extracellular space protein locations as compared to the low-fertility bulls. These transcripts include protamine 1, casein beta 2 and thrombospondin receptor CD36 molecule [14-16]. These studies shall pave way for elucidating transcriptomic changes associated with abnormal development in spermatogenesis and facilitate the improvement of assisted reproductive technologies and also serve as fertility markers [17,18], however, such study on fertility-related genes needs to be validated under field conditions which need enormous cost and manpower.

## Seminal Biomarkers

Breakthroughs in proteomics and genomics have led to the identification of various biomarkers in various biological fluids including semen for predicting fertility. Some of the semen biomarkers associated with fertility are fertility associated antigen, CATSPER family proteins, cysteine-rich secretory proteins, A-kinase anchor protein 4, cluster-of-differentiation antigen 9, cytochrome P450 aromatase, cathepsin D, α-l-fucosidase, phospholipase A2 bovine seminal plasma proteins, spermadhesin Z13, clusterin, osteopontin, heparin/gelatin binding proteins, and PDC-109-like protein [19-21]. These proteins are critical for normal sperm motility and male fertility. The capability to identify bulls on the basis of these fertility markers could result in higher pregnancy rate, leading to increased calf crop [22] nevertheless, these studies involving proteins is at the incipient stages along with the huge cost investment and sophisticated instrumentation for validation and to be used under field conditions.

## Ovum Pick Up (OPU)

This is a non-invasive and repeatable technique used for recovering large numbers of competent oocytes from antral follicles of live animals. This procedure was first done in cattle by Galli *et al*. [23], which was later tried in other species as well. Embryo production from ovum pick-up oocytes is affected by age, season, follicle stimulating hormone (FSH) stimulation and can average 1-3 embryos developed from oocytes collected per session [24]. It also evident that repeated OPU can be performed without side effects both in cattle and buffaloes with a minimal stress to the animal [25]. In India, the first buffalo calf (Saubhagya) was produced through this technique by Prasad *et al*. [26], and subsequently, first bovine calf (Holi) was produced at ICAR-National Dairy Research Institute. OPU has advantage to collect oocytes from animals with less invasiveness and the use of superior animals as oocyte donors in embryo transfer [27]. This method not only increases the lifetime reproductive efficiency but also can be used in follicle ablation for aiding follicle turnover during embryo transfer protocol. Follicular aspiration also allows studying the molecular intricacy and the role of various cytokines during folliculogenesis. One of the limitations of this technique is the low oocyte yield per ovary and necessity for sophisticated instrument for carrying out this technique [28,29].

## *In Vitro* Maturation, Fertilization and Culture (IVMFC)

This involves oocyte collection from slaughterhouse ovaries or from live animals followed by maturation and fertilization *in vitro* for the production of viable embryos. The first calf produced from *in vitro* fertilization (IVF) technique was in 1981 [30]. Various methods for *in vitro* maturation, IVF, and *in vitro* culture have been standardized in cattle and buffaloes with the production of calves worldwide [31,32]. In 1990 “Pratham,” the first IVF buffalo calf was produced in India [33]. In addition to *in vitro* embryo production (IVEP), development of various defined and semi-defined media for different species has been achieved. In addition, IVMFC has provided an excellent source of embryos for embryo transfer, cloning, transgenesis, and other advanced *in vitro* techniques. It has also allowed the analysis of the developmental potential of embryos, pattern of gene expression, epigenetic modifications and cytogenetic disorders in various domestic species and has been used as a model for human embryogenesis studies [8]. The main limiting factor of IVEP efficiency is low, i.e. 30-40% blastocyst development rate from oocytes after IVMFC [34]. This efficiency is much lower in buffalo, apart from lower recovery of oocytes in this species. The low success rate and the costs make the technique less feasible for application in buffalo as well in other livestock species under field conditions [35].

## Intracytoplasmic Sperm Injection (ICSI)

ICSI is a micromanipulation technique used for treating male infertility. It involves mechanical insertion of a selected sperm into the cytoplasm of an oocyte to produce desirable embryo. This technique helps in elucidating the different steps of fertilization. First mammalian egg injection procedure was reported by Lin [36] with the microinjection of golden hamster spermatozoa into hamster eggs. Since the first report of ICSI success, ICSI has been done in other species such as rabbits, mice, sheep, humans, horses, cattle, and pigs including buffaloes [37]. This technique is also used for sperm vector system for animal transgenic. ICSI has also been done with sexed semen with a success rate of 80% in cattle and 48-63% in small ruminants using fresh and frozen-thawed semen [32,38]. This technique involves steps, *viz*., preparation of oocytes and sperm for injection with oocytes collected from ovaries by aspiration. The collected oocytes are then examined, and sperm from frozen or fresh semen are prepared using the swim-up procedure, immobilized. Immobilized sperm is aspirated with tail first and injected into oocyte by positioning it against the holding pipette. The sperm is injected straight to the position of the polar body piercing the zona pellucida. Usually, it takes around 5 s for injecting sperm into the oocyte. Then, the oocytes are washed through a change of medium and then cultured overnight in suitable medium under mineral oil. The fertilized oocytes are examined 15-17 h postinjection for evidence of fertilization. This technique is of importance for studying the molecular pathways during the early stages of fertilization. ICSI in domestic species is similar to human which could be used as model training tool and in the use of sperm from superior bulls where the semen quality is compromised, nonetheless this technique requires sophisticated instruments and expertise for carrying out both under research and field conditions.

## Embryo Transfer Technology (ETT)

ETT is an important tool to improve livestock at faster rate as well as provides an opportunity to utilize the genetic contribution of both male and female [39,40]. ETT involves superovulation, an important step for increasing the number of oocyte from superior donors [41]. The transfer of mammalian embryos was first achieved by Walter Heape in 1890. Subsequently, progress in embryo transfer has been reported in various domestic species [31,35,42]. The birth of the first calf through embryo transfer was achieved by Betteridge [32]. Although superovulation in buffalo started three decades ago [43], the first live calves from bubaline embryos were born in 1983 in the USA and later in India [27]. Studies on superovulation among buffaloes have been carried out both in the river and swamp buffaloes in various countries including India [43,44]. Several protocols for superovulation of cattle and buffaloes, i.e. the concept of multiple ovulation and embryo transfer (MOET) were established in 1987 [45]. It was evident that MOET programs could result in increased selection intensity and reduced generation intervals, resulting in increased genetic gain. Superovulation is carried out in donors using hormonal preparations [39], mainly follicle stimulating hormones purified from porcine pituitaries (FSH-P) or pregnant mare’s serum gonadotrophin (PMSG). The response of PMSG was more but had adverse effect on the embryo recovery hence FSH preparations were preferred. On superovulation, there is increased in follicles and release of numerous ova from multiple follicles and hence double insemination with semen from superior bull is to be carried out. This is followed by flushing of embryos on day 6/7 in cattle and on day 5/6 in buffalo. Two methods have been followed, i.e., surgical and non-surgical. Surgical flushing results in increased number of embryos as compared to non-surgical but the former is more time and labor consuming and usually done in small ruminants [43]. For non-surgical flushing, Foley’s or Rusch catheter is used and suitable flushing medium is flushed in and out of the uterus to harvest the embryos at appropriate time post-estrus. Once the embryos are recovered, they are evaluated for features such as compactness, degree of degeneration, and suitability for transfer to the recipients programed along with the donors or else can be cryopreserved for future transfer. The conception rate following embryo transfer in cattle and buffaloes is around 35-45%, and the main limiting factor for the ETT is that this technique involves costly hormones, labor intensive protocols and expertise in addition to the poor super ovulatory response and pregnancy outcomes, especially in buffaloes.

## Embryo Cryopreservation

The mouse embryo was the first to be cryopreserved. For cryopreservation of embryos, conventional equilibrium methods involving freezing machines with controlled cooling rates and glycerol as cryoprotectant are usually followed. However, pregnancy rates obtained after transfer of the cryopreserved embryos are low [46-49]. To counter this issue, vitrification, introduced by Rall and Fahy [50] has replaced the conventional cryopreservation protocol. This method involves the use of highly concentrated aqueous solution of cryoprotective agents, *viz*., glycerol, ethylene glycol, and non-permeating agents such as sucrose, glucose, and fructose during no freezing equipment and hence considered superior as compared to slow freezing [50]. Vitrification has been used successfully for cryopreservation of cattle embryos at various developmental stages with the recovery rate of 88-89%. This technique is advantageous as it reduces the risk and expense in the transportation of expensive animals; reduce disease transmission and conservation of endangered species germplasm but the survival rate of frozen embryos diminishes thereby causes poor pregnancy rates following embryo transfer [51,52].

## Embryo Sexing

Embryo sexing is a technique in reproductive biotechnology having practical applications. Various procedures for embryo sexing have been used, *viz*., biopsy or cells aspiration. For this technique, embryos are collected on day 7 and are washed in buffer saline. Only excellent or good embryos are biopsied or aspirated. Usually, 8-10 cells are collected [53]. Thus, the total time required for sex determination is around 5 h. Sex determination is performed by Y-chromosome-specific DNA probe technology coupled with polymerase chain reaction (PCR) amplification of specific Y-chromosome region. Other methods involve detection of embryonic H-Y antigen in the embryos and use of loop-mediated isothermal amplification and duplex PCR-based assay showing more than 95% accuracy [54-56] but involves high cost, time and expertise for carrying out these protocols.

## Embryogenomics

This involves the study of genes’ expression in various developmental stage embryos in natural and altered environmental conditions through novel biotechnological tools [57]. By these methods, cascade of events during embryonic genome activation and development can be studied pertinent for early differentiation, successful implantation and fetal development [58]. Techniques such as differential display reverse transcription-PCR, subtractive cDNA libraries, quantitative real-time PCR, and microarray for studying transcriptomics have significantly extended the possibilities of revealing differences in mRNA expression patterns throughout the pre-implantation embryo development in various species. Various studies on the expression of marker genes, *viz*., glucose transporter Type 1, heat shock protein HSPA-1A, MATER, zygote arrest 1, growth differentiation factor 9, leukemia inhibitory factor, and bone morphogenetic protein 15 embryos/oocyte have been carried out [59-62]. Analysis of the genes will aid in selecting markers for determining quality embryo and be useful to assess the embryo normalcy and optimize assisted reproductive technologies [63-65]. However, the discovery of these markers needs to be validated under field conditions in addition to other genes which might contribute to embryo growth which needs enormous financial investment and expertise.

## Somatic Cell Nuclear Transfer

Somatic cell nuclear transfer also termed as “cloning” involves utilization of micromanipulation technique and cell fusion to transfer blastomeres of multicellular embryo or somatic cell into enucleated oocytes [66]. In this technique, nucleus of blastomere is reprogrammed for development of new embryo. It is useful technique used for multiplication of elite animals with minimal genetic variation [67,68]. Animal cloning was used for propagation of valuable genotypes, induce genetic modifications, and for producing transgenics. The first animal obtained by somatic cloning was a sheep - “Dolly” [69]. Since then, cloning has been done in various animals such as cattle, pig, goat and horse, besides buffalo and camel [24]. Cloning procedure using embryonic stem (ES) cells is called nuclear transfer-derived ES cell. Numerous types of somatic cells are used as donors in somatic cloning, *viz*., fetal fibroblasts, adult fibroblasts, granulosa cells, hepatocytes, lymphocytes [69,70]. Several studies have been reported for optimization of cloning procedures especially with production of handmade cloned embryos [70,71]. Successful pregnancy and production of cloned calves, *viz*., “Garima-I,” “Garima-II,” “Mahima” female calf were born from cloned buffalo “Garima” and male calves, “Shresth” and “Swaran” have been produced in India using handmade cloning technique [72,73]. Recently, a male cloned calf “Hisar Gourav” was produced at our institute, ICAR-CIRB, Hisar from adult somatic cells of tail obtained from progeny tested bull of superior genetic merit. Cloning holds the promise of bypassing conventional breeding procedures by allowing the creation of thousands of duplicates of genetically engineered animals. It can be used for the conservation as well as propagation of endangered species. It may be used as a tool for the production of stem cells for therapeutic cloning. This technique has opened novel opportunities for genetic engineering, animal genetic diversity conservation, tissue regeneration, and development of targeted ES cells for therapeutics. This technique can also be used in local breeds containing genes that confer adaptation, heat tolerance and disease resistance. In future, the scope of cloning can be in xenotransplantation, as it would allow multiplication of humanized animal models for research and therapeutics [24].

## Stem Cell Technology

Stem cells comprise those cells which have the capacity to become the progenitor of several other types of cells in the living system. These stem cells belong to the unspecialized or undifferentiated cells and retain the ability to become specialized cells under favorable or induced conditions. Based on their potency, stem cells are classified into different types, *viz*., totipotent stem cells, i.e. the cells derived from fertilized egg and can differentiate into embryonic and extra-embryonic cell types. The other type namely pluripotent stem cells are the descendants of totipotent cells and can differentiate into cells of the three different germ layers. On the other hand, multipotent stem cells can produce only closely related cells types [74,75] and unipotent stem cells are those which give rise to only one cell type, but possess the property of self-renewal. Likewise, based on their source, stem cells have been classified into three types, *viz*., embryonic, adult and fetal stem cells. ES cells are derived from embryos at a developmental stage before the time of implantation in the uterus. This is usually during the blastocyst stage (32 cell stage), and these ES cells can give rise to cells from all three embryonic germ layers As they do possesses the pluripotency markers, i.e., Oct4, Nanog and Sox2. The ESs cells are advantageous as they do not form tumors when transferred into the body which potentiates their use in transplantation. On the other, adult stem cells are those undifferentiated cells found throughout the body which is needed for replenish and regenerate cells in any damaged tissue. Such types of stem cells are found in bone marrow, neural, adipose and olfactory adult stem cells. In addition, there are fetal derived from fetal origin, i.e., amniotic fluid, umbilical cord and blood [76]. These cells are safe and reliable and have been used extensively in the biomedical application as they possess precursors of all differentiation lineages. And can be stored for long-term storage without change in their characteristics. These stem cells are a great promise in regenerative medicine both in human and livestock species and their usage in laboratory animals can be considered as model for the study of human ailments. However, the main limitations of using these cells is that the development of stem cell lines is robust along with the lack of cell-specific markers along with the lack of methods, at present for long-term culture and of pluripotency maintenance. In addition, it is also imperative to address the ethical concerns for the use of these cells both of diagnostic and therapeutic in livestock species [77,78].

### Transgenics

Transgenics involve transfer of gene using various methods within the genome for producing transgenic animals. The transgenic animals carry recombinant DNA within their genome, introduced by intervention [79]. The transferred gene consists of two parts: A functional part and a promoter region for carrying out function in the transfer host. Transgenesis has been carried out in various animals such as mouse, pig, sheep, goat, and cattle [80]. Efforts have been made in buffalo using ES cell-like cells isolated from *in vitro* fertilized and cloned blastocysts [72]. Several methods have been used for gene transfer, *viz*., pro-nuclear microinjection, retrovirus-based vectors, cytoplasmic microinjection, transferring DNA to embryos or ES cells via retroviral vectors, sperm-mediated gene transfer and RNA interference. Transgenic animals can be used both in breeding and also bioreactors [81]. Transgenic animals are used for producing animals’ models for disease resistance, mastitis resistant and for improved quantitative and qualitative traits [82]. Another use of transgenic farm animals is as bioreactors for producing human recombinant proteins in mammary gland [83,84]. As bioreactors, transgenic animals can be designed to produce milk of human growth hormone, high casein content, and human hemoglobin, anti-thrombin III and anti-tripsin in milk [82] and higher production of wool. In transgenic, pigs are used in xenotransplant studies [27]. This technology has also been used for transgenics model to understand various physiological in animals. Limitation factors include need for sophisticated instrumentation, cost and expertise in addition to the very low pregnancy success following constructed embryo transfer due to in appropriate genome editing [85,86].

### Nanotechnology

Nanotechnology is a recent advancement in the understanding of cellular and molecular biotechnology as well as its applications in various branches of biology including reproduction. This technology allows researchers to handle and study biological materials, i.e., cells, fluids in minute quantities. In addition to other field such as cellular biology, biotechnology, therapeutic medicine and genetics, it can also be another useful technique in farm animal reproduction [80]. In reproduction, microfluidics and nanofluidics [87] are recent tools to simplify traditional procedures of IVF and IVEP. More recently, reports have demonstrated the utility of microfluidics in isolation of motile sperm livestock. Oocyte manipulation under *in vitro* condition is also feasible with this technique [88]. Microfluidics can be used in sorting of sperm and embryos [89]. These systems control the flow of liquids or gases through a series of micro and nanoscale, which are in turn, controlled by computer circuited systems which aid in data analysis as well. Furthermore, development of biosensors for several markers has paved the way for identifying elite breeders and screen out genetic diseases. In farm animal, use of such sensors has been used in heat detection with nanotube under the skin to detect the changes in the level of estradiol in the blood. Biosensors for bovine milk progesterone with detection limit between 0 and 5 ng/ml have been developed. In addition, estrogen biosensor can detect to a limit of 10-150 pg and the detection limits were 1-2 ng and 1-1.8 ng for FSH and LH, respectively using the hormone specific biosensors [90]. The main limitation, at present with the use of this technology is the need of sophistication for developing these biosensors. In addition, designing of such sensors for various biomolecules across various species warrants enormous cost and time.

## Challenges and Future Prospective

The role and importance of recent biotechniques are steadily increasing due to the demand for increasing the productivity. However, these techniques are challenged by many factors, *viz*., lack of database on indigenous livestock and its biodiversity including production, reproduction, disease resistant traits within species and breeds necessary for the implementation of these techniques. Similarly, infrastructure, high cost and inaccessibility of these techniques to the stake holders are other factors preventing the harness of full potential of these techniques. In addition, a lack of expertise and interface between public and private partnership also adds up to the lesser reach of these techniques. Furthermore, apart from these discussed biotechniques, other allied fields, *viz*., genomics, proteomics, bioinformatics, and other “omics” are already been used in various filed of reproduction including livestock species. The use of these advanced techniques can further provide insight to the molecular intricacies of reproductive process including its derangement, in future. Therefore, there is an urgent need to have clear policy for proper adoption of these proven technologies and rendering them to the stake holders which require a multi-institutional approach to address the problems in animal production and reproduction.

## Conclusion

Reproductive processes in animals offer numerous advantages and scope for the use of novel biotechniques which were discussed in length in this review. Nevertheless, these emerging techniques should be judicially supplemented with good practices in animal health, nutrition and management at the level of stake holders for manipulation and improvement of health, production and reproductive performance of any livestock species, which will facilitate the production and dissemination of superior germplasm thereby enhancing the overall productivity of livestock species.

## Authors’ Contributions

KKC, KKM and AJ conceived the idea and collected information pertinent to the review. AJ and RKS drafted and edited the manuscript. All authors read and approved the manuscript.
